# Surgery scheduling heuristic considering OR downstream and upstream facilities and resources

**DOI:** 10.1186/s12913-020-05555-1

**Published:** 2020-07-23

**Authors:** Rafael Calegari, Flavio S. Fogliatto, Filipe R. Lucini, Michel J. Anzanello, Beatriz D. Schaan

**Affiliations:** 1 Department of Industrial Engineering, Federal University of Rio Grande do Sul, Av. Osvaldo Aranha, 99, 5° andar, Porto Alegre, 90035-190 Brazil; 2 Department of Critical Care Medicine, Cumming School of Medicine, University of Calgary, 3330 Hospital Dr NW, AB, Calgary, AB T2N 4N1 Canada; 3 Endocrinology Division, Hospital de Clínicas de Porto Alegre / Federal University of Rio Grande do Sul, Av Ramiro Barcelos, 2350, 4° andar, Porto Alegre, 90035-903 Brazil

**Keywords:** Surgery scheduling, OR sequencing, Break-in-moment, Surgical theater management, Operating room

## Abstract

**Background:**

Surgical theater (ST) operations planning is a key subject in the healthcare management literature, particularly the scheduling of procedures in operating rooms (ORs). The OR scheduling problem is usually approached using mathematical modeling and made available to ST managers through dedicated software. Regardless of the large body of knowledge on the subject, OR scheduling models rarely consider the integration of OR downstream and upstream facilities and resources or validate their propositions in real life, rather using simulated scenarios. We propose a heuristic to sequence surgeries that considers both upstream and downstream resources required to perform them, such as surgical kits, post anesthesia care unit (PACU) beds, and surgical teams (surgeons, nurses and anesthetists).

**Methods:**

Using hybrid flow shop (HFS) techniques and the break-in-moment (BIM) concept, the goal is to find a sequence that maximizes the number of procedures assigned to the ORs while minimizing the variance of intervals between surgeries’ completions, smoothing the demand for downstream resources such as PACU beds and OR sanitizing teams. There are five steps to the proposed heuristic: listing of priorities, local scheduling, global scheduling, feasibility check and identification of best scheduling.

**Results:**

Our propositions were validated in a high complexity tertiary University hospital in two ways: first, applying the heuristic to historical data from five typical ST days and comparing the performance of our proposed sequences to the ones actually implemented; second, pilot testing the heuristic during ten days in the ORs, allowing a full rotation of surgical specialties. Results displayed an average increase of 37.2% in OR occupancy, allowing an average increase of 4.5 in the number of surgeries performed daily, and reducing the variance of intervals between surgeries’ completions by 55.5%. A more uniform distribution of patients’ arrivals at the PACU was also observed.

**Conclusions:**

Our proposed heuristic is particularly useful to plan the operation of STs in which resources are constrained, a situation that is common in hospital from developing countries. Our propositions were validated through a pilot implementation in a large hospital, contributing to the scarce literature on actual OR scheduling implementation.

## Background

Hospitals are widely acknowledged as complex systems particularly in terms of management, with the surgical theater (ST) playing an important role, demanding a large portion of hospital resources and directly influencing patients’ flows [1, 2]. Several negative impacts may result from a poor ST management; most notably large queues of patients waiting for procedures, delays and cancelling of surgeries [3], and excessive working hours [4]. To avoid them, ST managers are challenged to synchronize the use of shared resources, such as operating rooms (ORs), surgical kits, post anesthesia care unit (PACU) beds, and surgical teams (surgeons, nurses and anesthetists) [5–7].

Block scheduling (BS) is an approach used to manage STs in many hospitals; e.g. [1, 5]. In BS, the availability of ORs is divided in time blocks, which are assigned to surgical specialties. The assignment is cyclic, varying according to demand and planning strategy. BS management is implemented in two main phases: (i) Master Surgery Scheduling (MSS) and (ii) Surgical Case Sequencing (SCS). Phase (i) is devoted to capacity planning, in which surgical specialties are assigned to one or more ORs considering a cycle (e.g. week or month). The objective is to define a surgery timetable to be used in a predefined planning horizon such that the weekly number of surgeries performed is maximized and waiting periods are minimized [8]. Phase (ii) is devoted to surgery scheduling in the time blocks considering the expected availability of PACU and inward beds as well as conflicts in the use of shared resources; e.g. surgical trays and equipment [9]. In this phase, surgeries are scheduled considering planning horizons varying from 24 to 48 h. Our work focuses on the second phase, being directed to the scheduling of elective surgeries.

Several authors propose surgery scheduling models aimed at improving the performance of STs [2, 10, 11]. In general, they search for the maximum assignment of surgeries to ORs without compromising service quality indicators. Specific objectives include maximization of patient throughput [12], and minimization of ST costs [13], waiting times of patients and surgical teams [14], makespan [15], deferral and postponement [16, 17], underutilization and overutilization of ORs, inward, intensive care unit (ICU) and PACU [18]. Mathematical approaches for the ST scheduling problem are also diverse; however, analysis of scenarios through stochastic programming [19], the use of simulation [20] and mathematical programming (linear, integer and mixed) [21] are predominant in the current literature. Regarding the scope of proposed models, articles may be divided into those that consider only the OR [22] and those that include available resources (e.g. surgical instruments and equipment) and supporting areas (e.g. PACU and ICU) in the modeling [18].

Regardless of the large body of literature on SCS [2, 6, 8, 11], some research gaps appear as opportunities. Few studies propose SCS models that include all resources involved in the operation of STs; i.e. models that consider the integration of the OR downstream and upstream facilities and resources [4, 11]. In addition, the majority of studies validated their propositions using simulated data or simplified ST instances. Complete implementation of SCS models in highly complex STs, reporting drawbacks and required assumptions and simplifications, are still missing in the literature. Real case applications of SCS in complex scenarios are key to determine the applicability of theoretical propositions, and are an important contribution to the state-of-the-art on the subject [23].

We aim at bridging those gaps in the literature by proposing a heuristic for surgery scheduling that takes into account the availability of ORs, materials required to perform the procedure, and PACU beds. The heuristic is grounded on the concept of Break-in-Moments (BIMs – time moments when surgeries are completed in an ST) [24] to maximize the number of surgeries performed while minimizing the variance of intervals between surgeries’ completions. Validation of our proposed heuristic is performed twofold: (i) comparing our suggested sequence of surgeries to past sequencing available in the database of a large University hospital, and (ii) through a 10-day pilot run at the ST of that same hospital, in which all relevant aspects of the practical implementation of our SCS heuristic are discussed.

## Methods

The problem of sequencing surgeries and synchronizing the use of resources in an ST is similar to what is known in several areas as the hybrid flow shop (HFS) [25] scheduling problem. HFS is used in production systems in which products or tasks are processed in more than one stage, such that in at least one of the stages there are machines operating in parallel. At each stage there is at least one machine that performs part of the processing required towards the final product. Each machine is able to handle one product/task at a time, and each product/task is processed by only one machine at each stage. Translating the HFS to an ST, each task (surgery) is assigned to a single machine (OR) belonging to a group of parallel machines in stage 1, and to a single machine (PACU bed) from a group of parallel machines in stage 2 [4]. There is a known processing time for each task as well as additional resources, which must be taken into account when generating the final schedule.

### Database

The database used in this study was made available by Hospital de Clinicas de Porto Alegre (HCPA), a high complexity tertiary University hospital located in the south of Brazil. There are 843 inward beds at HCPA, and a total of 32,000 patients are hospitalized yearly. HCPA’s ST is comprised of 15 ORs (one of which is dedicated to emergency surgeries) and performs average 11,680 surgeries per year. HCPA’s Ethical Committee has approved the study and authors have complied with the recommendations of the Declaration of Helsinki.

The database contained historical information on 71,175 surgeries performed between 2009 and 2014, covering 2681 different procedures. All procedures were mapped regarding surgical kits (instruments, wrapped and sterilized, needed to perform the procedure) and equipment demanded, and times related to them; namely: mean and standard-deviation of the occupancy time in the OR, and mean and standard-deviation of length-of-stay in the PACU. Information on procedures’ times were not directly available in the database, which carried the following temporal marks for each surgery entry: T_1_ – patient is retrieved; T_2_ – patient is prepared; T_3_ – patient enters the OR; T_4_ – patient is anesthetized; T_5_ – surgery starts; T_6_ – surgery ends; T_7_ – patient leaves the OR; T_8_ – patients enters PACU; T_9_ – patient leaves PACU. OR occupancy time was defined as the difference between T_7_ and T_3_; length-of-stay in the PACU was defined as the difference between T_9_ and T_8_. Table 1 presents a partial view of the time information for the 5 most frequently performed procedures.

**Table 1:** Five most frequently performed procedures at HCPA

Specialties	Procedure	N° of records	APD	SDPD	ARD	SDRD
Digestive tract	Laparoscopic cholecystectomy	2265	136	41	298	192
Urology	J double catheter placement	1827	77	29	372	272
Orthopedics / Traumatology	Hip Arthroplasty	838	201	45	390	215
Digestive tract	Exploratory laparotomy	816	145	54	538	348
Urology	Transurethral resection of the prostate	748	101	29	304	145

*APD* average procedure duration, *SDPD* standard deviation of procedure duration, *ARD* average recovery duration, *SDRD* standard deviation of recovery duration

A matrix **M**_0_ was created carrying information on surgical kits and equipment (column entries) required to perform procedures (row entries). Each cell (*i*, *j*) informed whether resource *j* was needed to perform procedure *i*, being assigned values 1 (yes) or 0 (no). Based on historical information from the sterilization unit, a processing time of 180 min was assumed to sterilize any surgical kit. Analogously, the time needed to clean any OR after a surgery was set to 20 min. An end-of-shift slack of 20 min was also considered to account for unforeseen events that could delay surgeries scheduled for the next shift.

### Proposed heuristic

The heuristic proposed in this paper was programmed in MATLAB R2012b as a two-stage problem. In stage 1 surgeries are assigned to ORs; in stage 2 the availability of surgical kits and PACU beds is verified. Scheduling in stage 1 must consider the availability results in stage 2. The problem’s combinatorial nature makes it mathematically complex and potentially NP-hard [24]. With that in mind, the proposed heuristic is based on assumptions and restrictions that reduce its mathematical complexity and enables the determination of an optimal solution within reasonable computing time; they are:

i. Once started, a surgery cannot be interrupted;
ii. ORs, surgical kits and equipment are ready to use (sanitized/sterilized) at the beginning of each shift;
iii. ORs and equipment are sanitized immediately after surgery completion, and PACU beds immediately after patients are discharged or moved to ward beds;
iv. Surgeries must start and finish in the same shift, except for ORs in which the same surgical team will operate in the next shift;
v. Elective surgeries are assigned to the morning and afternoon shifts;
vi. Priorities assigned by surgical teams must always be respected;
vii. PACU patients occupy beds for one or more shifts; and
viii. It is not possible to perform surgeries from different specialties in a given shift in the same OR.

The occupation of ORs and PACU beds and the use of surgical kits and equipment at different time instants is informed in three matrices (**M**_1_, **M**_2_, **M**_3_). The OR occupation matrix (**M**_1_) is organized such that the list the ORs available for surgery assignment in the ST are presented in the rows and the time instants of a shift, in minutes (e.g. 360 columns for a 6-h shift), in the columns. The other two matrices follow the same structure, with PACU beds (**M**_2_) and surgical kits and equipment (**M**_3_) replacing ORs in the rows.

**Fig. 1:**
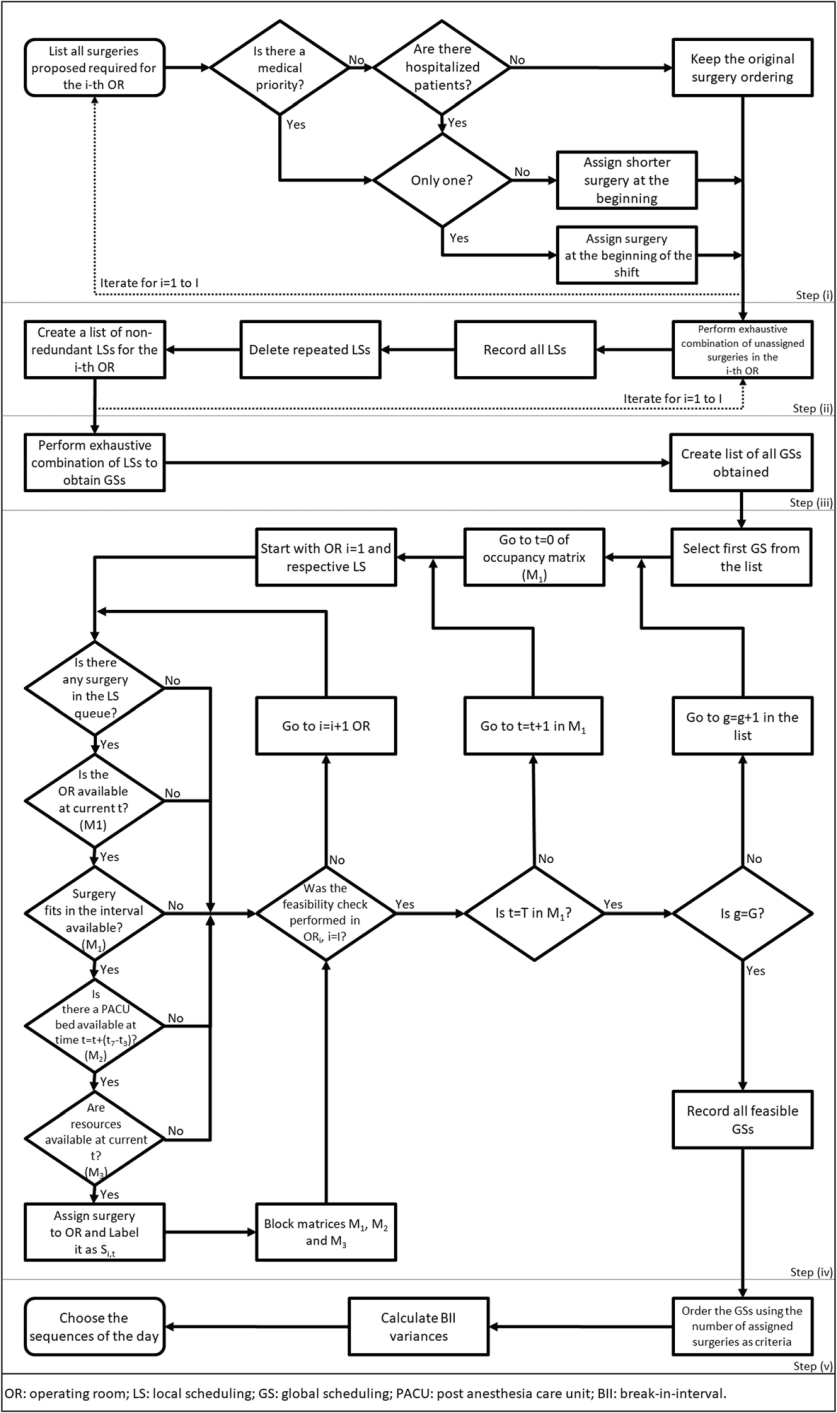
Flowchart with steps of the proposed heuristic

Our proposed scheduling heuristic is implemented in two stages and five main steps: (i) listing of priorities; (ii) local scheduling; (iii) global scheduling; (iv) feasibility check; (v) identification of best scheduling. Stage 1 is comprised of steps (i) to (iii); remaining steps integrate stage 2. The flow chart in Fig. 1 supports the description of the heuristic presented next.

In step (i) priority surgeries are identified for each OR and shift. Surgeries are considered priority whenever indicated by the surgical teams. Otherwise, we prioritize surgeries to be performed on hospitalized patients, minimizing the no-show probability. When more than one patient is hospitalized, we prioritize those to be submitted to surgeries with shorter estimated durations, since they tend to present shorter PACU lengths-of-stay. At HCPA, elective surgeries are not performed in the night shift; thus, prioritizing shorter surgeries will push longer procedures to the end of the morning and afternoon shifts, allowing patients to stay at the PACU overnight and optimizing its night occupancy. In addition, starting the morning shift with shorter procedures promotes early PACU occupancy, reducing its morning idleness. At the end of step (i), *OR*_*i*_ (*i* = 1, …, *I*) will have a list of surgeries to be performed on a given day. Besides the operational benefits derived from including such restrictions, they promote a significant reduction in the problem’s mathematical complexity. That allows us to apply a method that explores all possible scheduling solutions in search of the optimum, something that would not be feasible in an NP-hard problem.

**Fig. 2:**
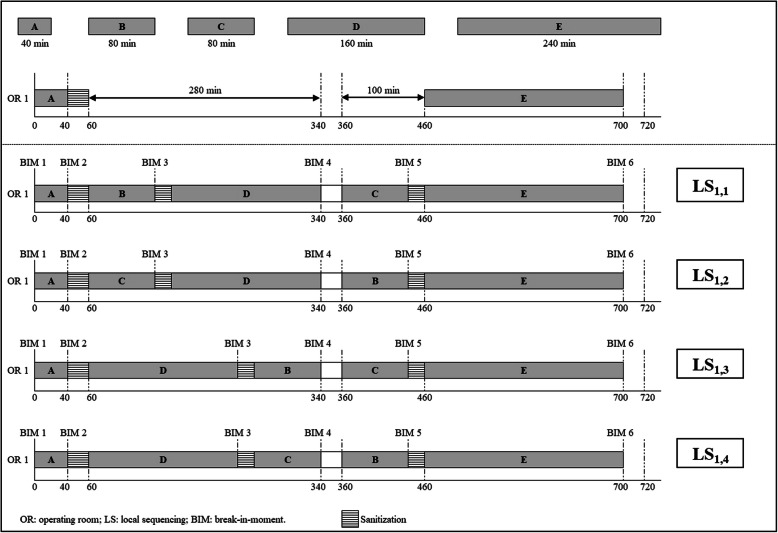
Example of local sequencing

In step (ii) all possible orderings (i.e. sequencing) of surgeries listed for *OR*_*i*_ and not prioritized in step (i) are enumerated; the procedure is repeated for *i* = 1 to *I*. At this point, resources and PACU bed availabilities are not yet considered. Each sequencing obtained for *OR*_*i*_ is denoted a *local sequencing* and represented by ${LS}_l^i$, such that *l* = 1, …, *L*. At the end of this step, a list of non-redundant LSs will be available for each OR. Figure 2 presents an example of LS considering an OR operating two 6-h (720 min) shifts with a list of 5 surgeries scheduled for that day (A, B, C, D, and E). Surgeries B and C are exactly the same procedures performed by the same team. The surgical team requested surgery A to be the first one performed and surgery E to be the last; remaining surgeries are iteratively assigned to remaining available time slots. There are four possible LSs in this example (${LS}_l^i$, where *i* denotes the OR and *l* the local sequencing number): ${LS}_1^1$, ${LS}_2^1$, ${LS}_3^1$ and ${LS}_4^1$. Next, duplicated LSs (i.e. those in which the same two procedures are performed on the same day in the same OR) are identified and treated as a single LS. In the example, ${LS}_1^1$ and ${LS}_2^1$, and ${LS}_3^1$ and ${LS}_4^1$ are duplicated. The final list of LSs in the example has only two sequences. At the end of step (ii), all ORs will have a list of LSs.

In step (iii) all LSs are exhaustively combined to obtain a list of *global sequencings* (GSs). Each *GS*_*g*_, *g* = 1, …, *G*, is comprised of a set of *I* LSs. For example, in an ST with *I* = 2 (*OR*_1_ and *OR*_2_) each GS will be comprised of two LSs. If we assume each OR with two non-redundant LSs (${LS}_1^1$, ${LS}_2^1$, ${LS}_1^2$ and ${LS}_2^2$) there will be a total of four possible GSs; namely: *GS*_1_ – ${LS}_1^1$ and ${LS}_1^2$; *GS*_2_ – ${LS}_1^1$ and ${LS}_2^2$; *GS*_3_ – ${LS}_2^1$ and ${LS}_1^2$; and *GS*_4_ – ${LS}_2^1$ and ${LS}_2^2$. At the end of step (iii) a list of *G* GSs will become available.

In step (iv) GSs listed in the previous step are submitted to a feasibility check. For that, we start by using matrix **M**_0_ to list materials needed in surgical procedures listed in the GSs; then, matrices **M**_1_, **M**_2_ and **M**_3_ are filled out as follows. Each GS contains one LS for each OR. Select the first GS from the list and go to time *t* = 1 in **M**_1_. Starting with the first OR (*i* = 1), verify whether: (i) there is any surgery to be scheduled in the queue of surgeries available for that OR [if so, that surgery will be submitted to checks (ii) to (vi)]; (ii) the OR is idle at current time *t*; (iii) the surgery fits in the interval available at the OR; (v) the surgical kit and equipment for the surgery is available at current time *t*; and (vi) a PACU bed will be available at the minute the surgery ends.

In case of having a negative answer to any of the above questions, no surgery is assigned to the first OR at *t* = 1 and we perform the feasibility check for the next OR at *t* = 1 and carry on, until the last OR is analyzed. Otherwise, the first surgery in queue is assigned to the *OR*_1_ in matrix **M**_1_, starting at *t* = 1 and lasting for the number of minutes corresponding to the mean duration of the surgery added of the time required to sanitize the OR once the surgery is finished. Additionally, in matrix **M**_2_ the patient is assigned to a bed at the PACU with stay starting at the minute the surgery ends, as noted in **M**_1_, and lasting for the number of minutes corresponding to the mean length-of-stay for that type of surgery at the PACU. Finally, in matrix **M**_3_ it is informed the length of time during which the surgical kit and equipment will be used in the surgery, added of the time required for sterilization, starting at *t* = 1. The surgery assigned to *OR*_1_ is removed from its queue and the next surgery becomes the first in queue.

Upon completing the checking at *t* = *T* for all ORs, all information on the current GS will be available in matrices **M**_1_, **M**_2_ and **M**_3_, and step (iv) is repeated for the next GS. At the end of this step a set of matrices **M**_1_, **M**_2_ and **M**_3_ will be available for each GS. Note that it is possible that a GS contains ORs to which only few surgeries listed in the ORs’ LSs have been assigned due to feasibility constraints. In that case, the GS is likely to display a small total number of assigned surgeries and will be penalized in the next step of the method.

In step (v) the best feasible GS is identified. For that, the set of GSs analyzed in step (iv) is ordered, from the GS presenting the largest number of assigned surgeries to the one with the smallest number. Then for the subset of GSs with largest number of assigned surgeries, calculate their respective BII variances. The best GS will be the one with the maximum number of assigned surgeries and the smallest BII variance. Note that a small BII variance will lead to a scheduling in which surgeries are likely to end at even-distanced intervals across the ORs. That is a desirable situation for a number of reasons: (i) promotes a better use of OR sanitization teams, minimizing waiting times; (ii) gives time to move equipment across ORs; and (iii) avoids demand peaks at the PACU and at the sterilization unit.

**Fig. 3:**
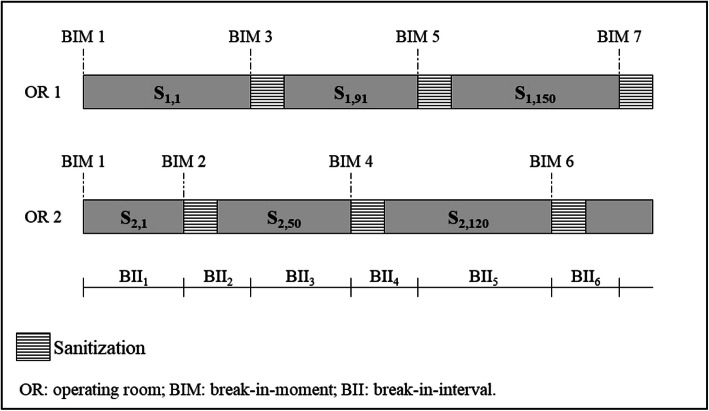
Determination of BIIs for an ST with two ORs

The calculation of a GS’ BII variance relies on the identification of its BIMs, as exemplified in Fig. 3 for the case of a ST with two ORs. The first BIM is defined as the start of the shift; remaining BIMs correspond to time periods in which surgeries are completed and patients are ready to be transported to the PACU. A BII is the interval between two consecutive BIMs; the variance of the BIIs is given by the variance operator for the sample of BIIs.

## Results

The proposed method was validated (i) comparing past scheduling of surgeries available in the database of HCPA with the scheduling proposed by our method (referred to as *test*), and (ii) running a 10-day pilot at HCPA’s ST (referred to as *pilot*) where surgical teams were invited to perform the surgeries following the sequence proposed by our heuristic. In both cases we considered 12 ORs (one OR is used exclusively for emergency procedures and other two were under renovation) and 15 surgical specialties; namely: General, Oral and Maxillofacial, Cardiac surgery, Digestive System, Pediatrics, Plastic, Thoracic, Vascular, Proctology, Gynecology, Mastology, Neurosurgery, Orthopedics, Oto-Rhino, and Urology.

### Test

The test was carried out using data from six typical days chosen by ST managers: 04/20/2015 (Monday), 04/23/2015 (Thursday), 05/04/2015 (Monday), 05/05/2015 (Tuesday), 05/08/2015 (Friday), and 05/12/2015 (Tuesday). We compared three scenarios: (i) the empirical schedule (ES) planned by each specialty’s surgical team; (ii) the actual schedule (AS) comprised of planned surgeries which were actually performed; and (iii) the scheduling obtained for the same group of surgeries considered in AS by our method, referred to as proposed schedule (PS). ES was exclusively based on expert opinion (i.e. surgical teams’ experience and preferences), with procedures’ durations determined by surgeons and not checked a priori for feasibility in terms of surgical kits and equipment needed, or PACU availability. PS corresponded to the GS with smallest BII variance among those with largest number of assigned surgeries. OR and PACU occupancies were used to compare the performance of each scenario.

Table 2 presents information on three performance criteria for each scenario: OR occupancy, number of surgeries assigned to ORs, and BII variance. Table 3 presents the results of paired sample *t*-tests run on pairs of scenarios for each performance criterion. In general, PS performed better than ES and AS over all criteria. Considering the average OR occupancy, PS promoted a 99%-significant improvement of 37.2% over AS. Analyzing the average number of procedures assigned to ORs, PS and ES displayed the same result (17.5 surgeries) which was 99%-significantly larger than the actual average number of surgeries performed in the ORs (AS) by 4.5 surgeries. Considering the average BII variance, PS and ES presented a value 95%-significantly smaller than AS, while no significant difference was found between PS and ES.

**Table 2:** Average occupation, number of procedures and variance of BIIs

Date	Average occupation				Number of procedures				Variance of BIIs			
	ES	AS	PS	I	ES	AS	PS	I	ES	AS	PS	I
04/20/2015	88.9%	66.7%	98.8%	**48.1%**	18	13	17	**4**	589.0	475.2	486.2	**−11.1**
04/23/2015	95.0%	77.2%	99.4%	**28.8%**	23	15	21	**6**	321.4	247.3	229.4	**18.0**
05/04/2015	96.4%	76.2%	99.7%	**30.8%**	15	10	16	**6**	341.2	2535.4	1093.2	**1442.2**
05/05/2015	89.6%	74.5%	95.2%	**27.8%**	15	12	15	**3**	4040.1	4614.4	2088.3	**2526.1**
05/08/2015	90.7%	70.6%	98.9%	**40.1%**	16	12	17	**5**	797.1	1379.3	763.4	**615.9**
05/12/2015	82.5%	62.3%	94.3%	**51.4%**	18	16	19	**3**	1065.4	2741.9	677.5	**2064.4**
**Average**	**90.5%**	**71.2%**	**97.7%**	**37.2%**	**17.5**	**13.0**	**17.5**	**4.5**	**1192.4**	**1998.9**	**889.7**	**1109.2**

*AS* actual schedule, *ES* empirical schedule, *PS* proposed schedule, *I* improvement (AS – PS)

**Table 3:** Results of paired sample *t*-tests

Scenarios	***t*** statistic	***p***-value	Conclusion
**OR occupancy**			
ES–AS	19.09	< 0.01	ES > AS
PS–ES	5.31	< 0.01	PS > ES
PS–AS	12.91	< 0.01	PS > AS
**Number of surgeries**			
ES–AS	5.32	< 0.01	ES > AS
PS–ES	0.00	0.50	PS = ES
PS–AS	8.00	< 0.01	PS > AS
**BII Variance**			
ES–AS	−2.10	0.045	ES < AS
PS–ES	−0.82	0.22	PS = ES
PS–AS	−2.54	0.026	PS < AS

**Fig. 4:**
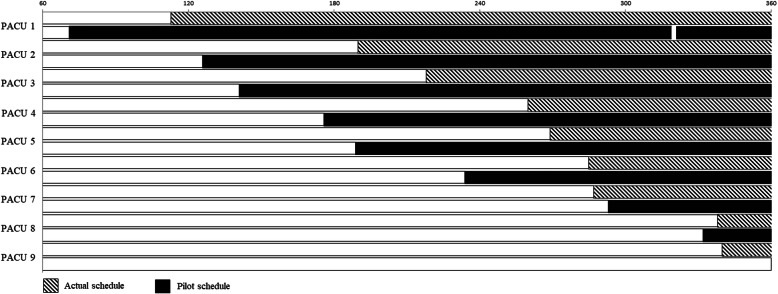
PACU bed occupancy

**Fig. 5:**
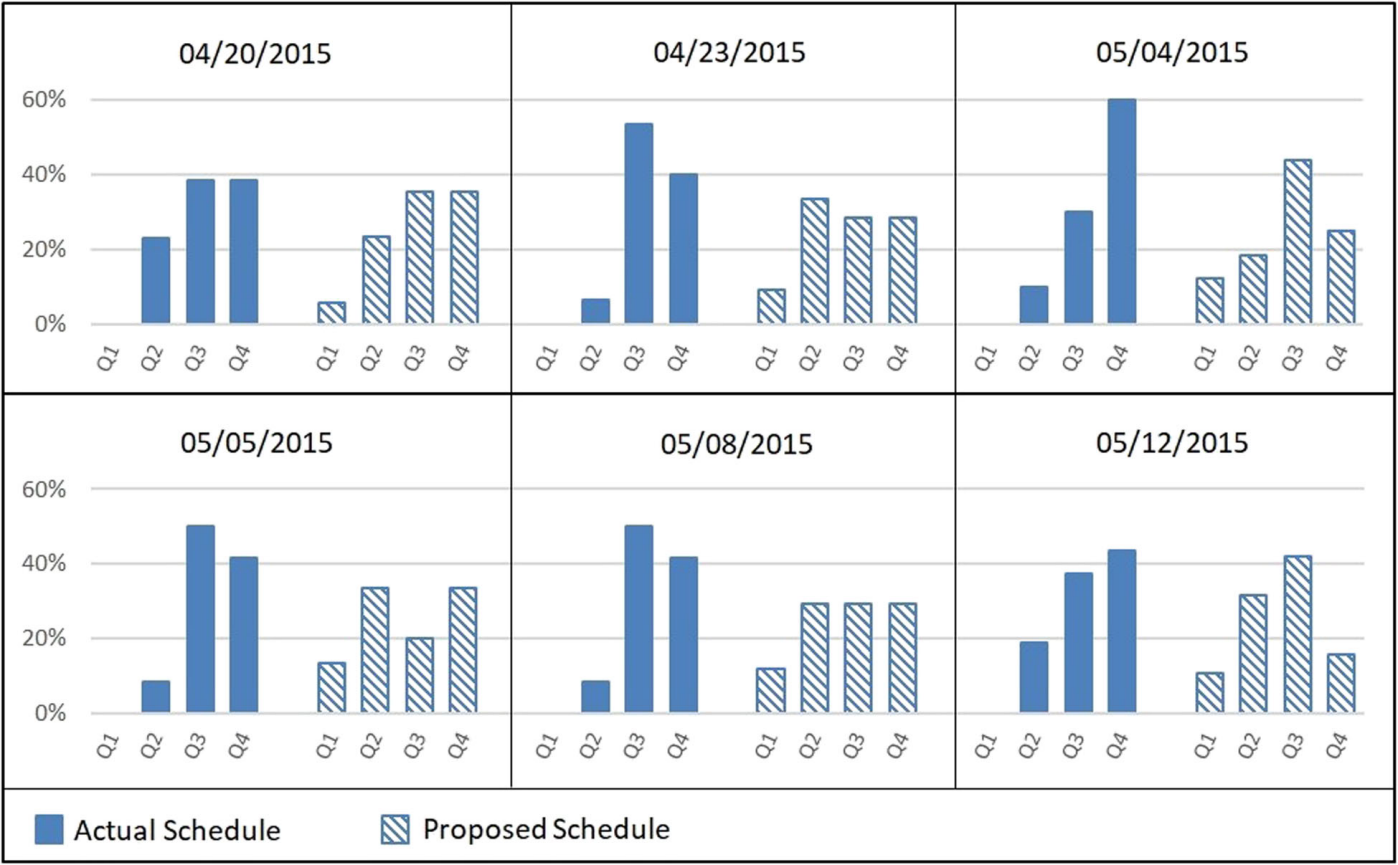
Distribution of PACU arrivals in shift quartiles under AS and PS

In addition to the three performance criteria above, we also evaluated the impact of our proposed schedule on PACU bed occupancy. Figure 4 presents the Gantt chart obtained comparing occupancies under AS and PS for one of the days simulated (05/08/2015). PS promotes earlier occupancy of PACU beds and a more uniform arrival of patients to the unit. The number of PACU beds required under PS is 8, one less than the number required under AS, since it promotes a turnover in PACU before the end of the second shift. These results reflect the benefits of choosing a GS with smaller BII variance and were observed on all other days tested. Figure 5 displays a comparison between actual schedule (AS) and proposed schedule (PS) with respect to patients’ arrivals at the PACU during shift quartiles (comprised of 90-min intervals), observed during six test days. It is visible that occupancy peaks present in AS were smoothed out in PS.

### Pilot

A pilot of the proposed method was run during ten working days at HCPA’s ST, from 07/20/2016 to 07/31/2016. It takes 2 weeks (i.e. ten working days) for all surgical specialties to rotate at the ST, since some specialties operate fortnightly. During the pilot, surgical teams from all specialties informed the surgeries to be performed on the next day by handing their ES to the team of researchers at 17 h00 of the previous day. Procedures in the specialties’ ES were rescheduled using our heuristic, and the proposed optimized schedules (PS) were informed back to the surgical teams.

Table 4 shows the results of the pilot. The average OR occupancy during the pilot was 78.19%, with average 28.5 surgeries performed each day, and average BII variance of 369.54. Regarding the number of surgeries, 29.12% of the ORs had a single surgery scheduled on a given 6-h shift and 30.81% of the ORs had two surgeries scheduled on a shift. Since we assumed that longer procedures would be scheduled last in the shift (unless otherwise specified by the surgical team), ORs with two surgeries per shift potentially display only one possible LS. With that, the complete enumeration process to determine the scheduling was run in 40.07% of the ORs presenting shifts with three or more surgeries scheduled.

**Table 4:** Results of the pilot test

Date	Occupation	Number of procedures	Variance of BIMs
07/20/2016	83.90%	30	356.23
07/21/2016	77.72%	30	355.74
07/22/2016	72.36%	29	224.51
07/23/2016	75.04%	26	421.88
07/24/2016	83.85%	23	1108.54
07/27/2016	80.42%	33	245.03
07/28/2016	81.26%	31	265.15
07/29/2016	74.11%	27	271.28
07/30/2016	77.32%	27	138.63
07/31/2016	75.93%	29	308.45
**Average**	**78.19%**	**28.50**	**369.54**

During the pilot, the day with smallest number of surgeries generated 24 GSs, and the computational time required to run our heuristic was 0.04 min. The day with the largest number of surgeries generated 165,888 GSs, demanding a computational time of 6.9 h. The median number of SGs was 4608, which required a computational time of 11.52 min.

## Discussion

According to the literature [24], surgery scheduling in large STs potentially leads to NP-hard combinatorial problems. However, our pilot test shows otherwise, since several surgical specialties are characterized by having long duration procedures (e.g. cardiovascular and neurological), which enable the assignment of a single surgery per shift (the problem exists even in cases of consecutive shifts occupied by the same specialty in the OR, since a change in the surgical team is expected). In the pilot, around 60% of specialties operated one or two surgeries per shift; as a consequence, a small number of GSs were generated when testing different assignment of surgeries to ORs, and the general scheduling problem could be solved by complete enumeration of GSs.

A key enabler of a feasible surgery scheduling is a good estimate of procedures’ duration [11, 14]. A common approach in STs is to ask surgical teams to estimate the duration of the time blocks required to perform surgeries [26]. Such estimates take into consideration the procedure to be performed, the patient’s health condition and the team’s expertise [26]. A study by Laskin et al. [27] shows that surgeons estimate correctly the duration of surgeries in approximately 26% of the cases, super estimate in 32% of the cases and sub estimate in 42% of the cases.

An alternative approach uses information gathered from electronic health records (EHR). Models based on EHR estimates perform better than those based on surgeons’ opinion [28]. For this reason, in our study we mined a database of surgeries performed between 2009 and 2014, and calculated the mean and standard-deviation (SD) of the duration of each procedure. Our proposed schedules considered surgeries with an estimated duration of mean + 1SD; this criterion was satisfactory during the pilot, during which no surgery occupied more than its estimated duration.

The pilot study was subjectively evaluated by the ST supporting staff, leading to the following observations. First, given that the BIMs of scheduled surgeries turned out be uniformly distributed, instruments and surgical trays to be sterilized arrive at the sterilization unit in a more balanced pace, allowing a close to optimal workload balance in the work shifts. Second, teams responsible for OR sanitization pointed out that the uniformly distributed times between surgeries’ completions not only allowed a better workforce scheduling, but also reduced the waiting time for the next surgery to take place in each OR. In HCPA there were only 2 sanitization teams and typically, nearly 20 min were required to prepare each OR for the next surgery. In case three ORs simultaneously had their surgeries concluded, one of them would have to wait 20 min until having its sanitization process started (i.e. that OR would have to wait 40 min until ready for the next surgery). Finally, the nursing staff reported having more time to prepare patients to enter ORs and move them after surgery to the PACU, minimizing queues.

The BIM concept was developed by van Essen et al. [24] with the objective of reducing waiting times of emergency surgeries. For that, the authors tested several heuristics aimed at minimizing the maximum distance between two consecutive BIMs. Using this strategy, the interval between surgeries became more evenly distributed along the day allowing emergency surgeries to be more quickly assigned to ORs. We based our method in their proposition; however, our objective was to minimize peaks in hospital resources’ demands and in patients’ arrivals at the PACU. We also proposed a new criterion to better distribute the interval between surgeries: the minimization of BII variance.

Among several aspects that characterize works with surgery scheduling propositions, three are noteworthy: (i) scheduling objective, (ii) resources and supporting areas considered, and (iii) scheduling validation. The objective of reducing resource demand peaks is known in the literature as leveling of resources, being already covered by other studies on surgery scheduling. Most authors aim at analyzing the impact of a given schedule on the use of resources within ORs, on patient waiting times, nursing staff workload and PACU bed occupancy [8, 11]. Marcon and Dexter [29], for example, used discrete event simulation to analyze the impact of assigning long or short surgeries first in the ORs on patients’ waiting times and arrival pattern at the PACU.

Due to the fact that an ST requires services and resources from different hospital areas the literature is diverse regarding scope and constraints considered in surgery scheduling models. Some authors focus on the impacts of scheduling on the surgery itself and on patients, disregarding resources’ availability [30, 31]. For example, Denton et al. [31] proposed and evaluated heuristics aimed at sequencing surgeries in light of uncertainties related to the length of surgeries. Heuristics were based on pairwise interchange, that takes advantage of lower bounds on the optimal solution, and on the use of mean and variance of surgery durations to select a sequence. Results indicated that assigning longer procedures at the start of shifts may significantly compromise OR performance measures. Other authors propose the integration of OR downstream and upstream facilities in the scheduling model; while some analyze variables related to PACU bed and resources availability, and the capacity of supporting areas individually [32, 33], others aimed at considering those variables simultaneously to best reproduce the operation of an ST [4, 13]. However, a reduced number of studies proposed scheduling models taking into account the availability of resources such as surgical kits, equipment and personnel in STs with limited resources.

Regarding the validation of scheduling models, the majority of studies analyze results obtained running the proposed model on historical or simulated data. The validation of models through simulation is relevant, allowing a controlled sensitivity analysis and highlighting important model characteristics; it does not guarantee however that the proposed models will be feasible in practice. Although evolving in recent years (e.g., [34]) scarce are the works reporting practical implementation of surgery scheduling models [11]. There are some reasons for that, most notably reaching an agreement with surgical teams regarding the proposed sequencing of procedures and managing unforeseen events that may occur in ORs.

In summary, we proposed a surgery scheduling heuristic that takes into account the availability of resources (surgical trays and equipment) and PACU beds to generate feasible schedules. Such heuristic is particularly useful to plan the operation of STs in which resources are constrained, a situation that is common in hospital from developing countries.

## Conclusions

To the best of our knowledge, this is the first study that proposes a surgery sequencing heuristic aimed at minimizing peaks in the use of ST resources, both upstream and downstream, searching for schedules that maximize the number of surgeries assigned to ORs while minimizing the BII variance. With this approach, it is possible to increase ST capacity with the same amount of resources (personnel and equipment). Based on simulated results, the proposed schedule performed better than the actual and empirical schedules over all criteria considered. Based on the pilot, we observed an increase in occupancy if compared to the actual schedule in the test. Our proposed heuristic considers actual restrictions that characterize large STs, which increases its potential use, and was validated through a pilot implementation in a large Brazilian hospital, contributing to the scarce literature on actual OR scheduling implementation.

The method was tested in a surgical theater whose operational restrictions allowed us to find the optimum scheduling by exhaustively exploring all feasible sequences. Not validating the heuristic in NP-hard instances is a limitation of the study. As future study, we envision an expanded heuristic that will include ambulatory procedures that share the same material resources in the scheduling. In addition, future studies should adapt our proposition to be used in surgical theaters with larger number of ORs, replacing the complete enumeration strategy by a suitable optimization heuristic.

## Data Availability

Based on a mutual agreement between researchers and Hospital de Clinicas de Porto Alegre (HCPA), the datasets used and/or analyzed during the present study are not publicly available.

## Abbreviations

ST: Surgical theater
OR: Operating room
PACU: Post anesthesia care unit
HFS: Hybrid flow shop
BIM: Break-in-moment
BII: Break-in-interval
BS: Block scheduling
MSS: Master surgery scheduling
SCS: Surgical case sequencing
ICU: Intensive care unit
HCPA: Hospital de Clinicas de Porto Alegre
LS: Local sequencing
GS: Global sequencing
ES: Empirical schedule
AS: Actual schedule
PS: Proposed schedule
HER: Electronic health records
SD: Standard-deviation
APD: Average procedure duration
SDPD: Standard deviation of procedure duration
ARD: Average recovery duration
SDRD: Standard deviation of recovery duration
I: Improvement (AS – PS)

## References

[1] Hovlid E, Bukve O, Haug K, Aslaksen AB, Von Plessen C (2012). A new pathway for elective surgery to reduce cancellation rates. BMC Health Serv Res.

[2] Guerriero F, Guido R (2011). Operational research in the management of the operating theatre: a survey. Health Care Manag Sci.

[3] Kaddoum R, Fadlallah R, Hitti E, El-Jardali F, El Eid G (2016). Causes of cancellations on the day of surgery at a tertiary teaching hospital. BMC Health Serv Res.

[4] Latorre-Núñez G, Lüer-Villagra A, Marianov V, Obreque C, Ramis F, Neriz L (2016). Scheduling operating rooms with consideration of all resources, post anesthesia beds and emergency surgeries. Comput Ind Eng.

[5] Santibáñez P, Begen M, Atkins D (2007). Surgical block scheduling in a system of hospitals: an application to resource and wait list management in a British Columbia health authority. Health Care Manag Sci.

[6] Hulshof PJH, Kortbeek N, Boucherie RJ, Hans EW, Bakker PJM (2012). Taxonomic classification of planning decisions in health care: a structured review of the state of the art in OR/MS. Heal Syst.

[7] Leeftink AG, Bikker IA, Vliegen IMH, Boucherie RJ. Multi-disciplinary planning in health care: a review. Heal Syst. 2020;9:95–118. 10.1080/20476965.2018.1436909 PMC7476549 32939255

[8] Samudra M, Van Riet C, Demeulemeester E, Cardoen B, Vansteenkiste N, Rademakers FE (2016). Scheduling operating rooms: achievements, challenges and pitfalls. J Sched.

[9] Pham DN, Klinkert A (2008). Surgical case scheduling as a generalized job shop scheduling problem. Eur J Oper Res.

[10] Gartner D (2015). Optimizing hospital-wide patient scheduling.

[11] Cardoen B, Demeulemeester E, Beliën J (2010). Operating room planning and scheduling: a literature review. Eur J Oper Res.

[12] Niu Q, Peng Q, ElMekkawy TY (2013). Improvement in the operating room efficiency using Tabu search in simulation. Bus Process Manag J.

[13] Neyshabouri S, Berg BP (2017). Two-stage robust optimization approach to elective surgery and downstream capacity planning. Eur J Oper Res [Internet].

[14] Pulido R, Aguirre AM, Ortega-Mier M, García-Sánchez Á, Méndez CA (2014). Managing daily surgery schedules in a teaching hospital: a mixed-integer optimization approach. BMC Health Serv Res.

[15] Dekhici L, Khaled B (2012). Operating theatre scheduling under constraints. J Appl Sci.

[16] Choi S, Wilhelm WE (2014). An approach to optimize block surgical schedules. Eur J Oper Res.

[17] Otten M, Braaksma A, Boucherie RJ. Minimizing Earliness/Tardiness costs on multiple machines with an application to surgery scheduling. Oper Res Heal Care. 2019;22:100194.

[18] Adan I, Bekkers J, Dellaert N, Vissers J, Yu X (2009). Patient mix optimisation and stochastic resource requirements: a case study in cardiothoracic surgery planning. Health Care Manag Sci.

[19] Bruni ME, Beraldi P, Conforti D. A stochastic programming approach for operating theatre scheduling. IMA J Manag Math. 2014:1–21.

[20] Cardoen B, Demeulemeester E (2008). Capacity of clinical pathways - a strategic multi-level evaluation tool. J Med Syst.

[21] Di Martinelly C, Baptiste P, Maknoon MY (2014). An assessment of the integration of nurse timetable changes with operating room planning and scheduling. Int J Prod Res.

[22] Agnetis A, Coppi A, Corsini M, Dellino G, Meloni C, Pranzo M (2014). A decomposition approach for the combined master surgical schedule and surgical case assignment problems. Health Care Manag Sci.

[23] Van Riet C, Demeulemeester E. Trade-offs in operating room planning for electives and emergencies: a review. Oper Res Heal Care. 2015.

[24] van Essen JT, Hans EW, Hurink JL, Oversberg A (2012). Minimizing the waiting time for emergency surgery. Oper Res Heal Care.

[25] Michael L. Pinedo. Scheduling - theory, algorithms and systems. 5th ed. New York: Springer International Publishing; 2016. p. 670.

[26] Tuwatananurak JP, Zadeh S, Xu X, Vacanti JA, Fulton WR, Ehrenfeld JM, et al. Machine Learning Can Improve Estimation of Surgical Case Duration: A Pilot Study. J Med Syst. 2019;43(3):44. 10.1007/s10916-019-1160-5 30656433

[27] Laskin DM, Abubaker AO, Strauss RA (2013). Accuracy of predicting the duration of a surgical operation. J Oral Maxillofac Surg.

[28] Wu A, Huang CC, Weaver MJ, Urman RD (2016). Use of historical surgical times to predict duration of primary Total knee Arthroplasty. J Arthroplast.

[29] Marcon E, Dexter F (2007). An observational study of surgeons ’ sequencing of cases and its impact on Postanesthesia care unit and holding area staffing requirements at hospitals. Int Anesth Res Soc.

[30] Bowers J, Mould G (2004). Managing uncertainty in orthopaedic trauma theatres. Eur J Oper Res.

[31] Denton B, Viapiano J, Vogl A (2007). Optimization of surgery sequencing and scheduling decisions under uncertainty. Health Care Manag Sci.

[32] Beliën J, Demeulemeester E (2008). A branch-and-price approach for integrating nurse and surgery scheduling. Eur J Oper Res.

[33] Beliën J, Demeulemeester E (2007). Building cyclic master surgery schedules with leveled resulting bed occupancy. Eur J Oper Res.

[34] Visintin F, Cappanera P, Banditori C, Danese P (2017). Development and implementation of an operating room scheduling tool: an action research study. Prod Plan Control.

